# Evaluation of Hyponatremia in Ischemic Stroke Patients in a Tertiary Care Hospital of Karachi, Pakistan

**DOI:** 10.7759/cureus.3926

**Published:** 2019-01-21

**Authors:** Sarfraz A Mahesar, Shehzeen F Memon, Sheema Mustafa, Amina Javed, Sara M Butt

**Affiliations:** 1 Neurology, Dow University of Health Sciences, Karachi, PAK; 2 Internal Medicine, Dow University of Health Sciences, Karachi, PAK; 3 Orthopaedics, Dow University of Health Sciences, Karachi, PAK; 4 Internal Medicine, Dow University of Health Sciences, Karachi, USA

**Keywords:** ischemic, stroke, hyponatremia, karachi

## Abstract

Introduction

Electrolyte disturbances are commonly found in acute stroke settings. Hypernatremia, hyponatremia and hypokalemia are the commonest types of electrolyte disturbances. Data on electrolyte changes in neurological disorders like stroke is insufficient in Asia. This study aims to quantify the decrease in sodium levels in patients of ischemic stroke and to see whether the presence of co-morbidities like hypertension and diabetes result in decrement in the sodium level of the patients admitted.

Methodology

This is a cross-sectional study conducted in Ruth M Pfau Civil hospital Karachi on 132 consenting patients diagnosed with ischemic stroke on a clinical and radiological basis using a preformed questionnaire with all the necessary information to evaluate the objective like gender, level of sodium and co-morbidities. The data was analyzed using Chi‑squared test using SPSS (Statistical Package for Social Science) software version 22 (IBM, NY, USA).

Result

The study showed that the majority of the patients (25%) had mild hyponatremia (130-134 mMol/L), only a few (9.8%) had moderate (125-129 mMol/L) or profound (<125) hyponatremia (3.8%) and 17 out of 44 patients who were hypertensive had their sodium levels changed while only 10 out of 17 diabetics had fluctuating sodium levels (p-value = 0.00). We also found out that most of the patients with altered sodium levels were male in the age range of above 55.

Conclusion

Patients with ischemic stroke do develop hyponatremia, but only with a slight alteration in the sodium levels.

## Introduction

Stroke is defined by the World Health Organization (WHO) as the clinical syndrome of rapid onset (usually seconds or minutes) of focal (or global, as in subarachnoid hemorrhage) cerebral deficit, lasting more than 24 hours or leading to death, with no apparent cause other than a vascular one [1]. Electrolyte disturbances may have negative influences on the outcome of acute phase of stroke and timely early detection and correction of dyselectrolytemia may improve the outcome of acute stroke. Electrolyte disturbance is commonly found in acute stroke settings, hypernatremia, hyponatremia and hypokalemia being a few common incidences amongst them. The disturbances usually result from the syndrome of inappropriate antidiuretic hormone (SIADH), increase of brain natriuretic peptides (BNP), inappropriate fluid intake and loss, and can result in seizures or death of the patient [2].

In patients with hyponatremia the total body sodium can be either increased, normal or decreased [3]. Hyponatremia is associated with an increased risk of death both during hospitalization and after hospital discharge. The in-hospital mortality among patients with this electrolyte change is greater than that among normonatremic patients [4]. The increased mortality among patients with hyponatremia appears to be attributable to progression of the underlying disease rather than to the electrolyte disorder itself, suggesting that hyponatremia may be a marker for more severe disease and a poor prognosis. Hyponatremia that is considered to be clinically asymptomatic may also be associated with falls and unstable cognitive abilities, as a result of which a person, along with being paralyzed, might also lose their cognition [5].

Data on electrolyte changes in neurological disorders like stroke is still tremendously scanty in third-world countries located in Asia, though India has surfaced to be a top researcher in this arena. Pakistan lacks quantification of such data in the form of research articles primarily because more emphasis is given on treating the disease than finding out the consequences or the changes that occur in a patient’s body while he is dealing with that ailment. Our study aims to quantify the decrease in sodium levels in patients of ischemic stroke and to see whether presence of co-morbidities like hypertension and diabetes result in decrement in the sodium level of patients admitted. The severity levels of hyponatremia used as a standard for our study have been added after a great deal of literature exploration. Joint European guidelines classify hyponatremia in adults according to serum sodium concentration as follows: mild (130-134 mMol/L), moderate (125-129 mMol/L) and profound (<125 mMol/L) [6].

## Materials and methods

This research is a cross-sectional study, conducted between the time period of February 2018 and June 2018 in Civil Hospital Karachi on established cases of Ischemic stroke diagnosed on the basis of clinical history, examination, and neuroimaging. A total of 132 patients were included in the study, who were admitted to different medical wards and neurological stroke unit. A pre-designed questionnaire was used to fill in required information for each patient. These included all necessary questions that would aid in building a relation to the eventual results, such as age, gender, clinical presentation, comorbid conditions, sodium levels and duration of symptoms. Sodium level of each patient who had stayed in the ward for at least 24 hours of admission was determined from the hospital records and no limitations were set on the day when the level of hyponatremia was noted except making sure that patients who were admitted for more than a week met our exclusion criteria because patients with a longer stay might have presented with an irregular decrease or might even get their levels normalized by a week's time and this might have created a bias if uniformity was not taken into account in considering the period when the levels were checked. Other electrolytes were not taken into consideration. Patients were divided into age groups of young adults, adults and elderly and full consent was gained from each one of them before proceeding. Bias was removed by having the data translated into local language. Patients who had had a previous stroke were excluded and only those with a first-time stroke were included in the study because patients with an old infarct might have presented with a pre-changed sodium level, resulting in ambiguity of results.

Compilation of the results was done and data was tabulated using SPSS (Statistical Package for Social Science) software version 22 (IBM, NY, USA). Frequencies of levels of hyponatremia were found using descriptive statistics. Chi-square was applied on categorical variables like levels of hyponatremia and co-morbidities in order to extract a relation between the two and the p-value came out to be 0.001, which showed statistical significance.

## Results

The results of this study conducted on 132 patients admitted with diagnosed ischemic stroke, showed that after a period of 24 hours, 51 patients had documented hyponatremia whilst 81 admitted patients did not develop hyponatremia, instead, a few contradictorily developed hypernatremia during their stay.

The primary objective of our study was to deduce the degree of hyponatremia and the type of hyponatremia occurring most commonly in the patients. The following Table 1 satisfactorily describes the results. According to the table, 33 (25% cases) had mild hyponatremia, 13 (9.8%) had moderate hyponatremia and only five (3.8%), in addition to these, had profound hyponatremia.

**Table 1:** Level of hyponatremia in patients.

Level of hyponatremia	Percentage of patients (Total = 100%)
Mild (130-134 mmol/L)	25%
Moderate (125-129 mmol/L)	9.8%
Profound (<125 mmol/L)	3.8%
No hyponatremia	61.4%

Table 2 shows that amongst the patients that also presented with co-morbidities, 17 out of 44 hypertensives and 10 out of 27 diabetics developed hyponatremia (p-value 0.001). Other co-morbidities studied were hepatitis and tuberculosis both being common in this part of the world, and ischemic heart disease as it is a common presentation in the same age when stroke usually occurs in a person and can lead to heart failure. Renal failure and liver failure were, however, not considered, though equally important in the development of stroke.

**Table 2:** Hyponatremia in patients with co-morbidities.

Co-morbidities	Hyponatremia present	Hyponatremia absent
Hypertension	17	27
Diabetes mellitus	10	17
Both	7	8
Others (Ischemic heart disease, Tuberculosis or Hepatitis)	7	20
None	10	9

Table 3 tells us that most of the patients were males (n = 79) in the ages between 56 and 75 (n = 43). Females were lesser in number in the same age group (n = 32) and also fewer in the younger age groups compared to their male counterparts.

**Table 3:** Age in relation with gender.

Age range	Males	Females
25-40	8	3
41-55	28	18
56-75	43	32
	Total = 79	Total = 53

As mentioned in Table 4, it was also noticed that males developed hyponatremia in a greater number compared to females. About 35 males were found to develop hyponatremia compared to only 18 females, the p-value came out to be 0.36, and as a result, no significant association could be built between the relation of patients’ gender with their hyponatremia.

**Table 4:** Hyponatremia according to genders.

Frequency (total = 132)	Gender	Hyponatremia
33	Male	Present
18	Female	Present
46	Male	Absent
35	Female	Absent

Conclusively, ischemic stroke patients did establish a decrement in their sodium level but most of them only had a slight alteration, a finding that has not been vastly covered in literature.

## Discussion

Hyponatremia in patients with an acute central nervous system (CNS) disease is the most common electrolyte disturbance encountered in neurological clinical practice [7, 8]. In our study, the motive was to find the severity of deviation of sodium level from the normal in ischemic stroke patients because literature proposes that sodium levels usually are not changed in patients observed for ischemic stroke compared to the patients admitted for hemorrhagic stroke, even though, patients with ischemic stroke did in reality present with decrement in sodium level whilst their stay in the Hospital. Out of the 51 patients in our study that presented with hyponatremia, the majority (25%) had mild changes in their sodium level while only a handful of them (3.8%) presented with profound hyponatremia.

In those who did have a decreased sodium level, hypertension was a registered co-morbid for which most of them were not taking medications probably due to their lower socio-economic status. Stroke incidence increases in men and women as arterial blood pressure, either systolic or diastolic, increases. Isolated systolic hypertension increases the risk of stroke in the elderly. Successful antihypertensive therapy decreases stroke incidence in asymptomatic persons, hypertensive patients with transient ischemic attacks and survivors of hypertensive stroke [9]. The decrement in sodium level of the patients due to these medications can be a good explanation for the development of hyponatremia. Older patients with diabetes have a high risk of vascular complications. They have an increased risk of approximately three times for developing stroke compared with subjects without diabetes. In a study, among stroke risk factors, the prevalence of diabetes mellitus was significantly higher among hyponatremic patients (p < 0.001) [10]. These patients have a higher mortality, worse functional outcome, more severe disability after stroke and a higher frequency of recurrent stroke. Likewise, having diabetes as a co-morbid was not a significant association with sodium changes in our study. Many of the patients had no co-morbidities whatsoever but 17 such patients did show an unstable sodium level.

Pradhan et al. [11] showed that out of 64 patients from 100, diagnosed for ischemic stroke, only six developed a change in sodium level with a mean level coming out to be 136 mMol/L. Our study displayed 33 out of 132 identified ischemic stroke patients developing hyponatremia with their sodium levels in the range of 130 mMol/L to 134 mMol/L. Al-Khazraji [8] showed that 17 out of a 100 patients with hemorrhagic stroke developed hyponatremia in the range of <135 mMol/L while no patients with ischemic stroke established a low serum sodium level, whereas in our study 32 (25%) patients were found to develop mild hyponatremia (<133-135 mMol/L). A factor contributing to dissimilar results could be a greater number of hemorrhagic stroke patients in their study and ischemic stroke being sole area of our exploration. Saleem et al. [12], however, did have results compatible to ours, though they had a much bigger sample population, about a 350. A total of 120 of the patients with ischemic stroke had changes in their sodium level; nonetheless, no quantification of the hyponatremia was done, as opposed to our study. In our study, we found the exact state of sodium levels in the patients admitted for at least 24 hours in the neurological ward but for not more than a week, 33 out of 51 cases had a decrement of only 2-3 mMol/L.

Electrolyte levels of patients should be kept in check from the moment they arrive because potassium and sodium are the chief electrolytes of the body and a switch in their levels can adversely affect the body on a mass level. Hyponatremia is an important cause of persistent altered sensorium in stroke patients [2]. It can also give various other neurological sign and symptoms like seizures, which can deteriorate the level of consciousness of the patient, hence, a quantification of the severity of hyponatremia needs to be done in order to have a clear vision about what levels can cause such adverse outcomes as mentioned above and deal with the problem in an orderly manner.

## Conclusions

Close monitoring of serum sodium levels helped us in finding the exact decrement level in patients of all ages and both genders and it was established that most of the cases only developed mild hyponatremia if they developed any, many of cases of ischemic stroke, however, did not develop hyponatremia at all. The evaluation of hyponatremia levels can help us in understanding the severity at which a patient can develop adverse outcomes in stroke, thereby helping to decrease the mortality rate.
